# Unexpected photosensitivity of the well-characterized heme enzyme chlorite dismutase

**DOI:** 10.1007/s00775-020-01826-8

**Published:** 2020-10-28

**Authors:** Durga Mahor, Julia Püschmann, Diederik R. Adema, Marc J. F. Strampraad, Peter-Leon Hagedoorn

**Affiliations:** Department of Biotechnology, Delft University of Technology, Van der Maasweg 9, 2629HZ Delft, The Netherlands

**Keywords:** Chlorite dismutase, *Azospira oryzae*, Heme enzyme, Stopped-flow spectroscopy, UV–visible illumination, Photosensitivity, Electron paramagnetic resonance, Oligomeric state

## Abstract

**Abstract:**

Chlorite dismutase is a heme enzyme that catalyzes the conversion of the toxic compound ClO_2_^−^ (chlorite) to innocuous Cl^−^ and O_2_. The reaction is a very rare case of enzymatic O–O bond formation, which has sparked the interest to elucidate the reaction mechanism using pre-steady-state kinetics. During stopped-flow experiments, spectroscopic and structural changes of the enzyme were observed in the absence of a substrate in the time range from milliseconds to minutes. These effects are a consequence of illumination with UV–visible light during the stopped-flow experiment. The changes in the UV–visible spectrum in the initial 200 s of the reaction indicate a possible involvement of a ferric superoxide/ferrous oxo or ferric hydroxide intermediate during the photochemical inactivation. Observed EPR spectral changes after 30 min reaction time indicate the loss of the heme and release of iron during the process. During prolonged illumination, the oligomeric state of the enzyme changes from homo-pentameric to monomeric with subsequent protein precipitation. Understanding the effects of UV–visible light illumination induced changes of chlorite dismutase will help us to understand the nature and mechanism of photosensitivity of heme enzymes in general. Furthermore, previously reported stopped-flow data of chlorite dismutase and potentially other heme enzymes will need to be re-evaluated in the context of the photosensitivity.

**Graphic abstract:**

Illumination of recombinantly expressed *Azospira oryzae* Chlorite dismutase (*Ao*Cld) with a high-intensity light source, common in stopped-flow equipment, results in disruption of the bond between Fe^III^ and the axial histidine. This leads to the enzyme losing its heme cofactor and changing its oligomeric state as shown by spectroscopic changes and loss of activity.
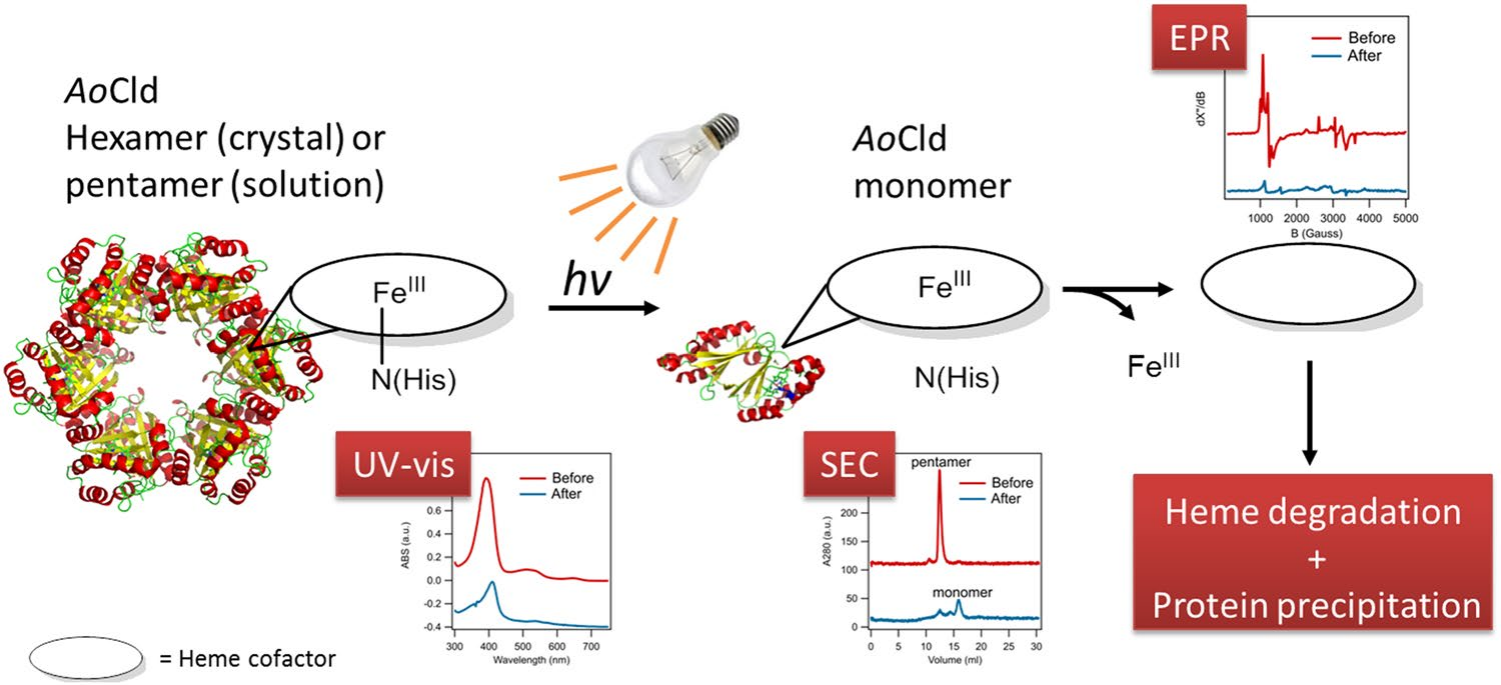

**Electronic supplementary material:**

The online version of this article (10.1007/s00775-020-01826-8) contains supplementary material, which is available to authorized users.

## Introduction

Chlorite dismutase (Cld) is a heme b-containing enzyme that converts the toxic chlorite (ClO_2_^−^) into harmless Cl^−^ and O_2_. Initially, it was identified in (per)chlorate-respiring bacteria; however, more recently, it has also been found in bacteria and archaea that do not have the ability to respire (per)chlorate [1, 2]. Most Clds exhibit extraordinarily high catalytic activity towards ClO_2_^−^ with turnover numbers in the range of 10^3^–10^4^ s^−1^ [3]. ClO_2_^−^ is the sole source of oxygen in the O_2_ product of the Cld reaction as shown by ^18^O labelling studies [4, 5]. The enzymatic O–O bond formation catalyzed by Cld is rare, as it is only the third known enzymatic system for such a reaction besides Photosystem II and the (not well characterized) nitric oxide dismutase [6, 7]. Interest in Cld has gained momentum as biotechnological applications have been developed, e.g., in wastewater treatment and controlled in situ oxygen production [1, 8–10]. Clds from different microbial sources have been recombinantly expressed, which made extensive biochemical and structural characterization of these enzymes possible [2, 11–16]. Recombinantly expressed Cld from *Azospira oryzae* (*Ao*Cld) is a homohexameric protein of which each subunit contains two ferredoxin-fold domains. One of the domains contains a heme b cofactor of which the iron is coordinated by an axial histidine H170. Close to the substrate binding site at the heme iron, a distal arginine R183 is positioned, which can adopt an “in” position, with the guanidinium group pointing towards the iron center, or an “out” position, with the guanidinium group pointing away towards a putative substrate channel. Furthermore, one or more of the four aromatic amino acids (Y118, W155, W156, W227), which are close to the heme, may play a role in electron transfer either as part of the mechanism or of an off-pathway [11, 13]. Despite the structural and biochemical knowledge of Clds, the reaction mechanism has not been fully established due to the high turnover rate of the enzyme with the natural substrate. Most reported pre-steady state kinetic information had been obtained using oxidizing agents like peracetic acid, hypochlorite and hydrogen peroxide using stopped-flow (SF) UV–visible spectroscopy experiments. SF spectroscopy of Cld from *Dechloromonas aromatica* (*Da*Cld) using peracetic acid (PAA) as an oxidizing agent showed sequential formation of compound I and compound II intermediates [4]. *Da*Cld compound I has been shown to oxidize typical substrates of peroxidase like guaiacol [17]. Similar intermediates were identified using SF spectroscopy of *Cyanothece* sp. PCC7425 Cld (*C*Cld) using hypochlorite as oxidizing agent [18]. The kinetics of CN^−^ binding has been reported for *Nitrospira defluvii* Cld active site and for *C*Cld in the time range from milliseconds to seconds [16, 19]. SF kinetic data of *Nitrospira defluvii* Cld versus chlorite have been reported in the time range of milliseconds to 11 s [20]. The spectral changes and heme bleaching were observed during chlorite turnover of this unusually slow chlorite dismutase (*k*_cat_ = 24 s^−1^ at pH 7). SF spectroscopic studies of *C*Cld versus hypochlorite and chlorite showed an apparent slow reaction of the enzyme that could not be part of the regular reaction mechanism, which was observable for more than 1 min [18]. This slow reaction was attributed to the chemical reactivity of highly oxidizing substrate intermediates that may be formed during the reaction, such as hypochlorite or chlorine monoxide. SF spectroscopy of *Da*Cld with excess hydrogen peroxide showed spectral changes and enzyme degradation in the time range up to 26 s [17]. Here, we demonstrate that *Ao*Cld is photosensitive during SF spectroscopy in the time range from milliseconds to minutes, which in part explains the previously observed slow spectral changes. Extended illumination of the enzyme with UV–visible light resulted in a loss of activity, spectroscopic changes of the heme cofactor, and changes of the oligomeric state of the protein.

## Materials and methods

### Chemicals and sample preparation

All chemicals were of analytical grade and purchased from Sigma. Horseradish peroxidase (HRP) and Bovine heart Cytochrome c (Cyt c, oxidized) were also purchased from Sigma. HRP and Cyt c concentrations were determined using a molar extinction coefficient *ε*_403nm_ = 100 mM^−1^ cm^−1^ and *ε*_550nm_ = 8.4 mM^−1^ cm^−1^, respectively [21, 22]. The proteins were dissolved in Milli-Q water to a final concentration of 1 mM and used for spectroscopic studies.

### Protein purification of *Ao*Cld

The *Ao*Cld expression strain (*E. coli* BL21(DE3) pLysS containing *Ao*Cld-pET28a-CDBC), which produced the codon-optimized and N-terminally his-tagged enzyme was previously described [23]. The *Ao*Cld DNA and amino acid sequences are given in the supplementary information. The *Ao*Cld expression strain was cultivated in TB medium containing 50 µg mL^−1^ kanamycin, 25 μg mL^−1^ chloramphenicol and 60 μg mL^−1^ hemin (pre-dissolved in 1.4 N NaOH). *Ao*Cld expression was induced with the addition of 0.5 mM isopropyl β-d-1-thiogalactopyranoside (IPTG) when the OD_600_ of the culture reached a value of 0.5 and the cells were subsequently cultivated at 25 °C for 7 h. Cells were harvested by centrifugation using a Sorvall centrifuge at 17,000×*g* for 10 min at 4 °C. The cells were washed and resuspended in 20 mM Tris–HCl pH 7.5 containing 500 mM NaCl, 50 mM imidazole, 1 mM PMSF, 1 mM MgSO_4_, 200 mg L^−1^ lysozyme and 10 μg mL^−1^ DNase. The cell suspension was disrupted by the Cell disruptor (CF1, Constant System Ltd.) at 1.5 kbar. The cell-free extract was obtained as the supernatant after centrifugation (Sorvall) at 16,770×*g* for 90 min at 4 °C followed by filtration (0.22 µm Steritop filter, Millipore). Enzyme purification was performed using IMAC chromatography using a Ni-Sepharose 6 Fast flow column (GE Healthcare) and an NGC medium pressure liquid chromatography system (Bio-Rad). All steps of enzyme purification were performed at room temperature (20 ± 2 °C). The cell-free extract was loaded on the Ni-Sepharose column that had been equilibrated with 20 mM Tris–HCl pH 7.5 containing 500 mM NaCl and 50 mM imidazole and a linear gradient from 0 to 100% 20 mM Tris–HCl, 150 mM NaCl, 500 mM imidazole pH 7.5 was applied. *Ao*Cld started to elute at circa 325 mM imidazole. All fractions of *Ao*Cld were pooled and desalted. Removal of excess imidazole and buffer exchange was achieved in two stages. First, desalting was performed using a HiTrap Desalting Column (GE Healthcare) equilibrated with 20 mM Tris–HCl pH 7.5. Second, buffer exchange was performed using a PD10 desalting column (GE Healthcare) equilibrated with 50 mM KPi pH 7.0. To remove bound imidazole from purified *Ao*Cld, the protein was extensively dialyzed against 100 mM KPi pH 7.0 at 5 °C. Protein purity was measured using UV–visible spectroscopy and SDS-PAGE. The theoretical Mw of the *Ao*Cld subunit is 30.3 kDa. Pure *Ao*Cld fractions were pooled and concentrated using an Amicon ultra-centrifugal filter (30 kDa Mw cut-off) by centrifugation at 3214×*g* at 4 °C for 20 min. Protein aliquots were frozen in liquid nitrogen and stored at − 80 °C. The protein concentration was determined by UV–visible spectroscopy (*ε*_280nm_ = 52.41 mM^−1^ cm^−1^) and using the Bicinchoninic acid (BCA) assay kit (Uptima, Interchim) with bovine serum albumin as the standard using the manufacturers’ instructions.

### UV–visible spectroscopy

UV–visible spectroscopic measurements were performed using the Cary 60 spectrophotometer (Agilent Technologies) at 21 °C. 10 µM of *Ao*Cld in 100 mM KPi pH 7.0, unless stated otherwise was used for measurements in a wavelength range of 200–800 nm.

### Chlorite dismutase activity assay

*Ao*Cld activity was measured polarographically by Clark-type oxygen electrode (type 5331, YSI Life Science) [24]. The electrode was calibrated with 100% air-saturated buffer and 0% oxygen by the addition of a reducing agent, sodium dithionite at 20 °C. The standard reaction mixture consisted of 1 mM sodium chlorite, 135 pM *Ao*Cld in 100 mM KPi pH 7.0 in a 3 mL reaction chamber.

### Measuring the photosensitivity of *Ao*Cld during stopped-flow spectroscopy

Pre-steady state kinetic measurements of *Ao*Cld were performed using the SX20 Stopped-flow (SF) spectrometer (Applied Photophysics). SF spectroscopic measurements were performed aerobically at 20 °C using a rapid scanning photodiode array, an optical quartz cell with a 10 mm pathlength and a 150 W xenon-arc lamp as light source. The light intensity of the SF setup was measured using Avasoft 8.11 with the IRRAD add-on software (Avantes) and an Avaspec 3648 spectrometer calibrated with an Avalight HAL-CAL-mini halogen lamp (Avantes). For the range 380–420 nm, the photon irradiance was 5.76 mE L^−1^ s^−1^ (57.6 mmol m^−2^ s^−1^ with a 1 cm pathlength). *Ao*Cld (20 µM) in 100 mM KPi pH 7.0 was mixed (1:1) in the SF with 1 eq. (20 µM) sodium chlorite in Milli-Q water and spectra were recorded every 60 ms for 60 s. In additional experiments, *Ao*Cld (20 µM) in 100 mM KPi pH 7.0 was mixed (1:1) in the SF with 1, 50, 500 and 5000 eq. (20 µM, 1, 10, 100 mM) sodium chlorite in Milli-Q water and spectra were recorded every 0.2 s for 200–250 s. As a control experiment, *Ao*Cld in 100 mM KPi pH 7.0 was mixed (1:1) in the SF with Milli-Q and spectra were recorded every 60 ms for 60 s. HRP (20 µM) and Cyt C (17 µM) in water were mixed (1:1) in the SF with Milli-Q water and spectra were recorded every 60 ms for 60 s. Each experiment was repeated three times, showing identical results. SF data of *Ao*Cld were analysed using singular value decomposition (SVD) with KinTek explorer software to generate kinetic traces and component spectra [25]. The reaction was modelled assuming an irreversible first-order mechanism:

$$E\mathop{\longrightarrow}\limits^{{k_{l} }}E_{{{\text{inact}}}}$$

### Measuring the effect of illumination on the spectroscopic and structural properties of *Ao*Cld

The light source was disconnected from the SF instrument. An optical bench, replicating the optical part of the SF, which was shielded from ambient light, was constructed. This allowed direct illumination, using the light guide (optical fiber) from the SF, of a 200 µL quartz cuvette with a path length of 10 mm containing 150 µM *Ao*Cld. The enzyme was illuminated for 1, 2, 5, 15, 30, 60 and 180 min. The cuvette was taken out from the set-up and UV–visible spectrum (after 15 × dilution) and the specific activity was measured as described above. The dependence of the specific activity on the illumination time was fitted to a biexponential decay curve which is described in the supplementary information. Samples for size-exclusion chromatography were produced by diluting 100 µL illuminated *Ao*Cld (30, 60 and 180 min) to 0.5 mL with 100 mM KPi pH 7.0. The samples were injected onto Superdex-200 10/300 GL column (GE Healthcare), pre-equilibrated with 100 mM KPi pH 7.0 and run at a flow rate 5 mL min^−1^ using an NGC medium pressure liquid chromatography system (Bio-Rad) at 20 °C. Chromatograms were recorded at 280 nm and 410 nm. The column was calibrated using the following molecular weight standards: 670 kDa (Thyroglobin), 158 kDa (Bovine γ-globulin), 44 kDa (Chicken ovalbumin), 17 kDa (Equine myoglobin), 1.35 kDa (Vitamin B_12_).

### Electron paramagnetic resonance

EPR spectra of illuminated and non-illuminated *Ao*Cld were recorded on a Bruker EMXplus 9.5 spectrometer. The low temperature was maintained by boiling liquid helium and cold helium vapor was passed through a double-wall quartz glass tube, which was mounted and fitted in the rectangular cavity [26, 27]. Illuminated samples were prepared as described above and diluted to 75 µM *Ao*Cld in 200 µL KPi pH 7.0. EPR tubes were frozen with liquid nitrogen and measured using the following conditions: Microwave frequency, 9.405 GHz, microwave power, 20 mW, modulation frequency, 100 kHz; modulation amplitude, 10 Gauss; temperature, 20 K.

## Results and discussion

### *Ao*Cld is photosensitive during stopped-flow spectroscopy

**Fig. 1:**
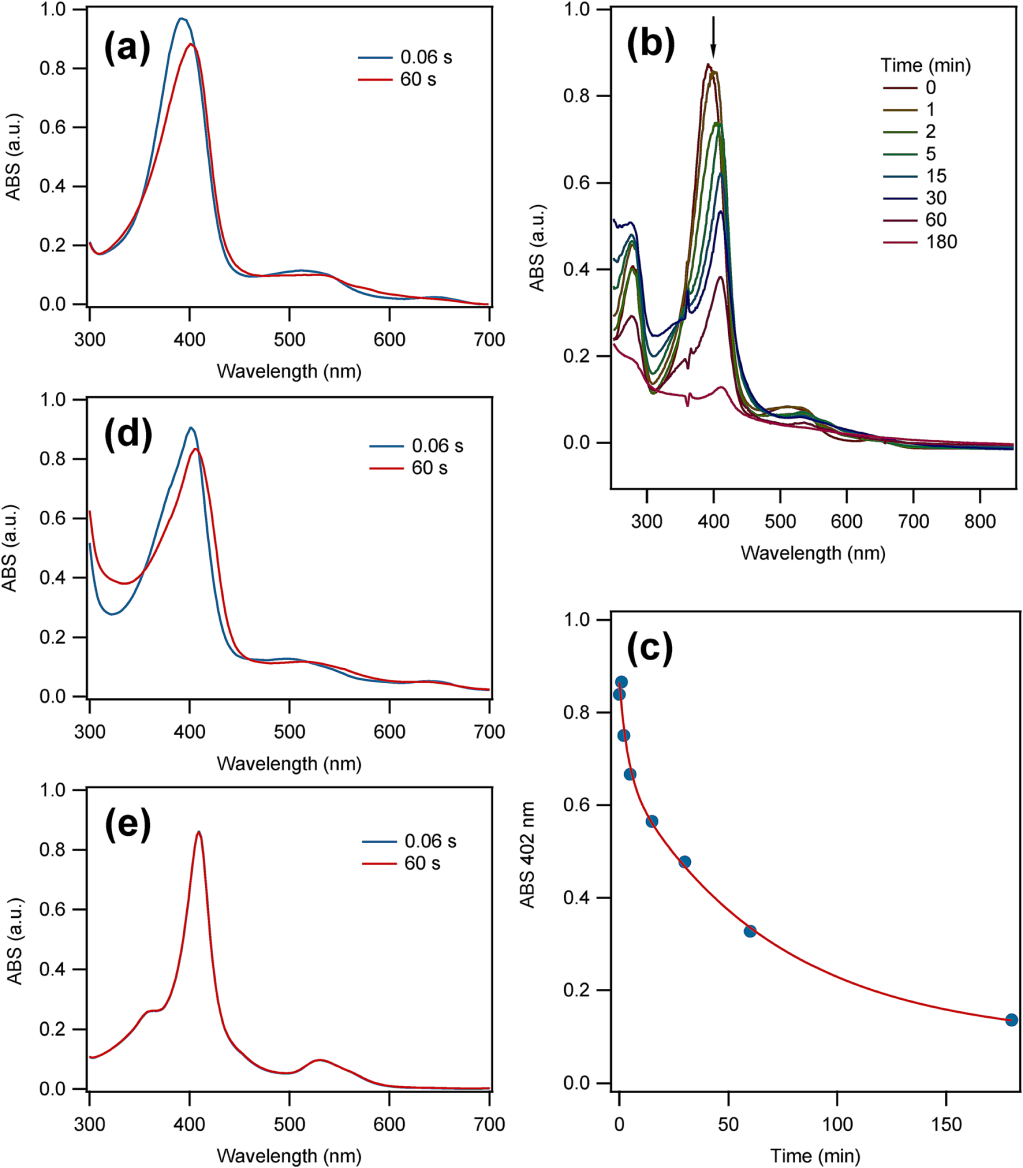
The effect of illumination of the stopped-flow (SF) light source on the UV–visible spectra of heme proteins. **a** SF spectroscopy of 18 µM *Ao*Cld in 100 mM KPi pH 7.0 against Milli-Q water in 1:1 ratio at 20 °C. The spectra at 0.06 s (blue) and 60 s (red) are given. **b** UV–visible spectroscopy of 16 µM *Ao*Cld in 100 mM KPi pH 7.0 mixed 1:1 with Milli-Q water at 20 °C after different illumination times by the Xenon arc lamp. **c** Decay of the Soret peak at 402 nm of *Ao*Cld during the illumination experiment under (**b**). The solid red line is a fit to a double exponential decay with a first-order rate constant *k*_decay,1_ = (0.43 ± 0.35)·10^–2^ s^−1^ and *k*_decay,2_ = (2.3 ± 1.2)·10^–4^ s^−1^ (details of the fit are given in the supplementary information). **d** SF spectroscopy of 20 µM Horseradish peroxidase in water against Milli-Q water in 1:1 ratio at 20 °C. The spectra at 0.06 s (blue) and 60 s (red) are given. **e** SF spectroscopy of 17 µM Bovine heart Cytochrome c (oxidized) in water against Milli-Q water in 1:1 ratio at 20 °C. The spectra at 0.06 s (blue) and 60 s (red) are given

During pre-steady-state kinetic analysis of *Ao*Cld and ClO_2_^−^ using SF spectroscopy, we observed significant spectral changes in the millisecond to the minute range (supplemental Fig. S2b and Fig. S3). These changes could not be attributed to the enzyme reaction, as the half-life time of ClO_2_^−^ was circa 2 ms under the conditions that were used. The same SF spectroscopy experiments with *Ao*Cld in the absence of substrate revealed identical spectroscopic changes (Fig. 1a and supplemental Fig. S2a and S2b), which shows that the spectral changes were indeed substrate-independent.

The UV–visible spectrum of ferric *Ao*Cld in 100 mM KPi pH 7.0 exhibits a Soret band at 391 nm, characteristic of the high-spin ferric 5-coordinate heme of the resting state enzyme. During SF spectroscopy experiments in which *Ao*Cld was mixed (1:1) with water, a redshift of the Soret band from 391 to 402 nm and a simultaneous decrease of the absorbance were observed (Fig. 1b). Prolonged illumination of *Ao*Cld from 1 to 180 min caused a further redshift from 402 to 409 nm and reduction of the Soret absorbance (Fig. 1b). Furthermore, the Q-band at 510 nm and the CT1 band at 647 nm disappeared, and a new band at 533 nm emerged together with a gradual loss of the heme absorbance. The redshift of the Soret band from 392 to 402 nm in the first minute of the illumination, precedes the time-dependent loss of the Soret absorbance. This suggests a photochemical modification of the heme environment, which results in the loss of heme over time. The reduction of the Soret peak follows irreversible double exponential decay of which the two phases are characterized by half-life times of circa 3 min and 50 min, respectively (Fig. 1c). Protein precipitation was observed after 60 min of illumination. Since the effects could not be attributed to the substrate ClO_2_^−^ as well as any buffer components and the effects were not observed during measurements in monochromatic spectrophotometers in the same time range (supplemental Fig. S1), we attribute the observed effects to the polychromatic illumination by the high-intensity light source (150 W xenon arc lamp) used in the SF instrument. For the range 380–420 nm, the measured photon irradiance of the SF setup was 5.76 mE L^−1^ s^−1^.

To investigate if the photosensitivity is unique to *Ao*Cld, the effects of the illumination on two canonical heme proteins, Horseradish peroxidase (HRP) and Cytochrome c (Cyt c), during the SF experiments were measured. HRP exhibited a similar redshift of the Soret band over time (Fig. 1d). Cyt C did not show any change of the UV–visible spectrum upon illumination (Fig. 1e). The photosensitive behavior of HRP under UV light has been reported previously [28, 29]. In HRP A2, illumination with UV and visible light caused direct or indirect oxidation of the axial histidine, which was responsible for the loss of enzyme activity and stability and resulted in changes in the electronic spectrum consistent with the release of the heme cofactor [28, 29].

**Fig. 2:**
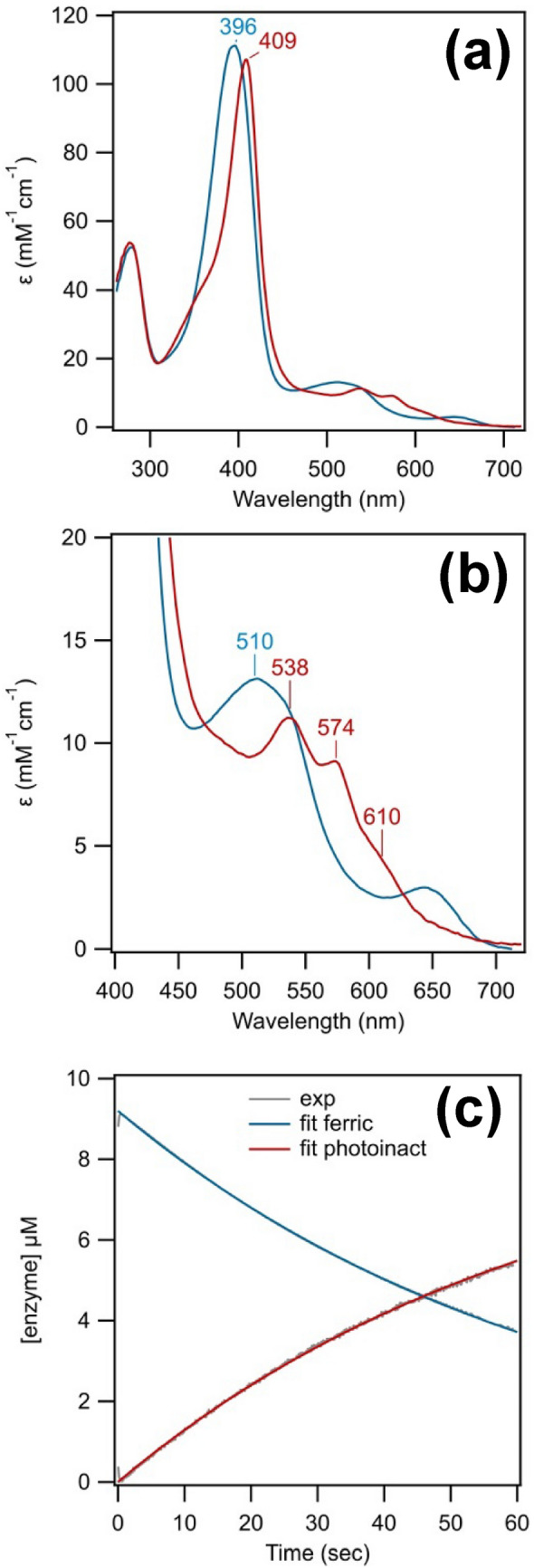
Kinetic analysis of SF spectroscopy of *Ao*Cld versus 1 equivalent (eq.) chlorite using singular value decomposition (SVD) assuming an irreversible first-order reaction. **a** Reconstructed spectra obtained after SVD analysis and fitting. In blue is the ferric resting state and in red is the photoinactived species. **b** Reconstructed spectra focussed on Q-bands. In blue is the ferric resting state and in red is the photoinactived species. **c** Kinetic traces in grey represent the reconstructed traces from the SVD analysis and the red and blue traces represent the fit to an irreversible first-order reaction with *k*_1_ = (1.51 ± 0.05)·10^–2^ s^−1^. SF experimental conditions: 18 µM *Ao*Cld in 100 mM KPi pH 7.0 against 18 μM sodium chlorite (1 eq.) in water in 1:1 ratio at 20 °C

Kinetic analysis of the SF experiments of *Ao*Cld versus water and various concentrations of chlorite was performed using singular value decomposition assuming an irreversible first-order kinetic model. The analysis resulted in the identification of the UV–visible spectrum of the photo-inactivated species (Fig. 2a,b and supplemental Fig. S2c,d) and the kinetic traces for the interconversion of the ferric resting state enzyme to the photo-inactivated species (Fig. 2c, supplemental Fig. S2e,f). The spectrum of the photo-inactivated species is characterized by a Soret band at 409 nm and Q bands at 538 and 574 nm, with a shoulder of at circa 610 nm that may represent a CT1 band. The spectrum indicates a mixed high-spin/low-spin population and is remarkably similar to the ferric hydroxo adduct, which is dominant at high pH (*pK*_*a*_ circa 8 in phosphate buffer) [30, 31]. The Q bands are characteristic of a low-spin ferric species, while the presumed CT1 band suggests a high-spin ferric component. The spectrum of the photo-inactivated species is also similar to the reported compound 0/III spectrum of *Da*Cld, which has a Soret at 408 nm and Q bands at 535 and 575 nm [17]. Compound 0 represents a ferric-hydroperoxo species, while compound III is a ferric-superoxide (or ferrous-oxo) species. This compound 0/III-like species formed transiently when *Da*Cld was mixed with a large excess of hydrogen peroxide. The first-order rate constant for the SF experiment of *Ao*Cld versus water was *k*_1_ = (1.82 ± 0.03)·10^–2^ s. This corresponds to a half-life time of 0.6 min. Clearly, the UV–visible spectroscopic change precedes the aforementioned decay of the Soret absorbance.

The same SF experiments were also performed with higher equivalents of chlorite, and at least up to 500 eq. (5 mM final concentration after mixing), the resulting spectra and kinetic traces were very similar (Supplemental Fig. S3). This means that the enzyme that has experienced zero up to 500 turnovers exhibits the same photochemical effect. Only at 5000 eq., the irreversible first-order kinetic model is insufficient to explain the results.

The known mechanisms for photo-induced damage to proteins involve the formation of electronically excited states via absorption by specific amino acid residues (tryptophan, tyrosine, phenylalanine, histidine, methionine, and cysteine) and cofactors (heme, flavin) sometimes associated with redox reactions leading to the formation of radical species and singlet oxygen [32]. Various studies describe the photosensitive nature of heme-containing enzymes, such as sunflower catalase, bovine liver catalase and HRP [28, 29, 33, 34]. In the case of catalase, which has an axial tyrosine, illumination at 365 nm resulted in UV–visible spectral changes of the heme, which were attributed to the formation of oxyferrous enzyme and compound II. A mechanism involving light-induced electron transfer from the ferric iron to the axial Tyr was proposed resulting in the oxyferrous state and a Tyr cation radical. One study proposed that photons are capable of aromatic amino acid ionization that stimulates heme bleaching [35]. Grotjohann et al*.* disclosed that blue light could cause heme dissociation and photochemical reactions in a plant catalase [33].

### *Ao*Cld subunit dissociation and cofactor instability during UV–visible light illumination

**Fig. 3:**
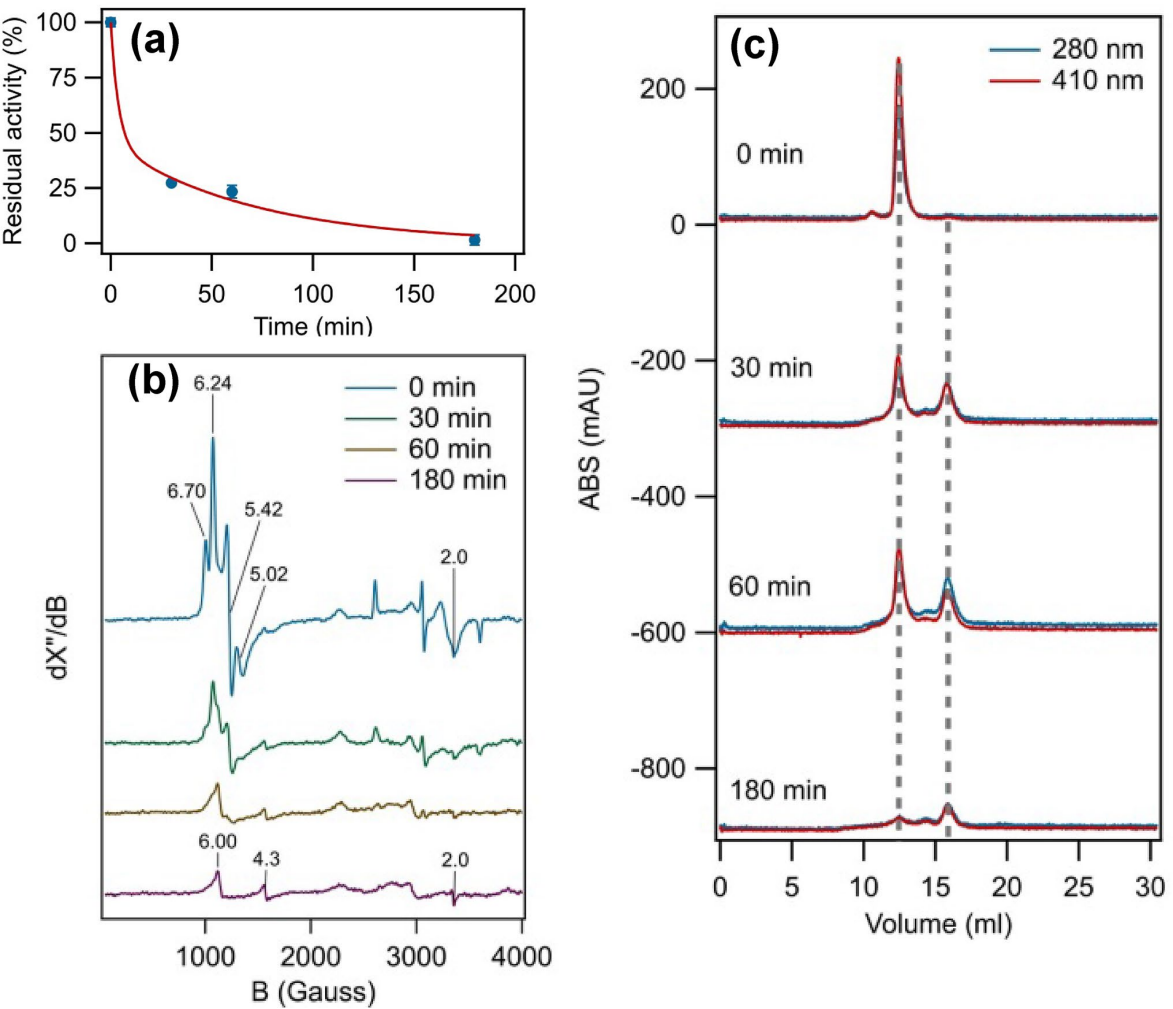
Characterization of *Ao*Cld oligomeric state, activity and EPR properties after illumination. **a** The specific activity of non-illuminated (0 min), 30 min, 60 min and 180 min illuminated 135 pM *Ao*Cld in 100 mM KPi pH 7.0 and 1 mM sodium chlorite at 21 °C. The red line is a double exponential curve with rate constants as determined for the Soret decay, with only the two amplitudes of the two phases as fitparameters. 100% activity represents a specific activity of (8.53 ± 0.17)·10^3^ U/mg **b** EPR spectra of non-illuminated and illuminated *Ao*Cld: 75 µM of non-illuminated (blue) and the same concentration of protein illuminated for 30 min (green), 60 min (brown) and 180 min (purple) prior to freezing. EPR conditions: microwave frequency, 9.405 GHz, microwave power, 20 mW, modulation frequency, 100 kHz; modulation amplitude, 10 Gauss; temperature, 20 K. **c** Analytical size exclusion chromatography was performed before illumination (0 min) and after 30 min, 60 min and 180 min illumination. All gel chromatograms were recorded at two different wavelengths 280 nm (blue) and 410 nm (red). Calibration of the column is given in supplemental Fig. S4

To characterize the effect of illumination on *Ao*Cld, enzyme activity measurements, EPR spectroscopy and size-exclusion chromatography (SEC) were performed on samples of the enzyme that were illuminated for 30, 60 and 180 min using the light source from the SF instrument. The enzyme exposed to illumination lost its activity in the timescale of minutes to hours. However, the non-illuminated enzyme remained stable. The non-illuminated *Ao*Cld had a specific activity of 8.53 ± 0.16·10^3^ U/mg, whereas illuminated samples (30 min, 60 min and 180 min) rapidly lost the specific activity by 72.6%, 76.6% and 98.5%, respectively (Fig. 3a and supplemental Table S1). The decay of the activity was consistent with the biphasic decay with the two rate constants observed for the Soret band (Figs. 1c, 3a). The UV–visible spectroscopic change observed in the SF experiments is characterized by a higher rate than those observed for the Soret decay and loss of activity, however, the data do not fully exclude the possibility that part of the loss of activity and the initial UV–visible spectroscopic change may occur simultaneously.

EPR spectroscopy reveals changes of the heme moiety upon illumination. The EPR spectrum of non-illuminated *Ao*Cld shows the characteristic combination of two axial high-spin ferric signals with *g*-values *g*_zyx_ = 6.24, 5.42, 2.0 and 6.70, 5.02, 2.0 (Fig. 3b) [30]. Varying populations of highly similar two high-spin species have been observed for Clds from other microorganisms (*Dechloromonas aromatica, Nitrospira defluvii, Magnetospirillum* sp., *Ideonella dechloratans*) [4, 12, 36, 37]. The precise nature of the two high-spin species remains to be fully established, but the ratio between the two species has been found to change depending on pH, buffer and in the presence of a high NaCl concentration. Several Clds, predominantly dimeric enzymes from non-perchlorate respiring bacteria (*Nitrobacter winogradski, Pseudomonas chloritidismutans, Klebsiella pneumoniae, Cyanothece sp.* PCC7425), exhibit a sharp axial high-spin ferric signal [5, 14, 16, 37]. Minor low-spin signals due to imidazole and hydroxide adducts are found with *g*-values *g*_zyx_ = 2.96, 2.25, 1.51 and *g*_zyx_ = 2.54, 2.18, 1.87, respectively [30]. After illumination at different time intervals, a gradual change of the high-spin signal to a single axial high-spin ferric signal with *g*_⊥_ = 6.00 and *g*_||_= 2.0, similar to free heme, is observed [38]. An isotropic signal at *g* = 4.3, which represents non-specifically bound ferric iron, increased with longer exposure times (Fig. 3b). Upon longer exposure times, the high- and low-spin signals diminished, which shows the degradation of the heme cofactor. The EPR results show that upon illumination the heme is released (or at least the axial ligand is released), with subsequent heme degradation and release of ferric iron. The spectra do not show clear radical formation, which suggests that no accumulation of radical species occurred.

The effect of illumination on the oligomeric state of *Ao*Cld was determined using SEC. The calculated molecular weight of *Ao*Cld is 30.3 kDa for the monomeric form. The non-illuminated sample was eluted as a predominant peak corresponding to a molecular weight of 140 kDa, which is consistent with the pentameric form of *Ao*Cld (Fig. 3c, supplemental Fig. S4). SEC of the enzyme after 30 and 60 min illumination showed two dominant peaks corresponding to 140 kDa and 21.3 kDa, and a minor peak corresponding to a molecular weight of 52 kDa (supplemental Table S2). This indicates dominant pentameric and monomeric forms of the enzyme, with a minor amount of dimer. The 180 min illuminated sample showed the same pentameric, dimeric, and monomeric states but with a different distribution in which the monomer is dominant (Fig. 3c). Interestingly, all oligomeric states clearly show absorbance at 410 nm, which shows that all the forms are heme-containing, although the lower *A*_410nm_/*A*_280nm_ ratio of the dimeric and monomeric forms suggests that these are partially heme-deficient. EPR spectroscopy showed more extensive heme depletion at 180 min than was observed with SEC. This could be due to precipitated protein that was present in the EPR samples, while only the remaining soluble protein was observed by SEC. The amplitudes of the peaks in the chromatogram of the 180 min illuminated sample were reduced compared to the other samples due to partial precipitation of the protein. It has been reported that the W155F variant of *Da*Cld is unstable at low concentrations, resulting in a loss of the pentameric state, changes in the secondary structure and heme loss [39].

### The photosensitivity may explain apparent slow reactions of Cld previously observed using SF

Several studies with pre-steady-state kinetic data that aimed to elucidate the catalytic mechanism of Cld have been published [4, 17–20, 40]. Because the enzyme has such a high *k*_cat_, the mechanism with the natural substrate has not been fully established to date. Studies with oxidizing agents, hydrogen peroxide and peracetic acid showed the production of high-valent Compound I and II species. Interestingly, at a timescale from milliseconds to minutes, too slow to be relevant for the catalytic mechanism, spectral changes were observed that were tentatively attributed to chemical effects of reactive chlorine species formed during the reaction and somehow escaped the mechanism [18]. Shaffner et al. report SF experiments of 1.5 μM *C*Cld versus 500 μM chlorite (333 turnovers) at pH 7.0 under conditions in which the complete conversion of chlorite takes circa 3.5 s. Fast spectral changes were observed within the dead time of the instrument, which may represent the first turnover. Subsequently, slow spectral changes took place after complete consumption of the substrate, which continued up to 80 s. We propose that these slow reactions are, in part, the result of the photosensitivity of Cld in SF experiments. Therefore, the previous reports on the catalytic intermediates and transient kinetics of Clds that have been obtained using SF spectroscopy with white light and a photodiode detector have to be revisited. It is most likely that the intermediates and rates that have been observed using artificial substrates represent a combination of Cld reaction intermediates and photodegradation products. As most of Clds are closely phylogenetically related, we may presume that most, if not all, Clds share the photolabile nature described here [2, 41]. SF spectroscopy using monochromatic light (single wavelength) most likely does not suffer from the photosensitivity reported here.

### The mechanism of Cld photo inactivation

Here, we present the effect of UV–visible light illumination on the activity, heme cofactor spectral properties, and the oligomeric state of *Ao*Cld. SEC and EPR spectroscopy provided evidence of heme dissociation during illumination. However, the precise mechanism remains elusive. We can only speculate on a possible mechanism in the absence of further spectroscopic evidence. One possible mechanism could involve one of the nearby tryptophans, W155 or W156, which upon photoexcitation could donate an electron to the heme cofactor, under the formation of a tryptophan cation radical. The one-electron-reduced heme could bind molecular oxygen forming a compound III, Fe(II)–O_2_, complex, similar to oxyhemoglobin or oxymyoglobin, which may explain the changes in the UV–visible spectrum. Subsequently, the Fe(II)–O_2_ complex can autocatalytically form a compound II species, Fe(IV)=O, and water. Degradation of compound II would further lead to bleaching of the heme and disruption of the oligomeric state of the enzyme. This suggests that the photoeffect present here would be oxygen-dependent, which is currently under investigation by us. All SF and illumination experiments reported here were performed under aerobic conditions. Possibly, a photochemical reaction of the heme group results directly or indirectly in the dissociation of the axial histidine, with subsequent heme release and a conformational change, which ultimately results in dissociation of the *Ao*Cld subunits and loss of activity. Alternatively, the photoexcitation of W155 could already trigger instability of the enzyme, in a manner similar to the W155F variant of *Da*Cld [39].

## Conclusion

Illumination of *Ao*Cld with UV–visible light resulted in changes in the UV–visible spectrum, indicating a possible involvement of oxyferrous and compound II intermediates during the photochemical inactivation. The EPR spectral changes indicate the loss of the heme and release of iron during the process. The oligomeric state of the enzyme changed from homo-pentameric to monomeric with subsequent protein precipitation. We attribute these effects to either direct or indirect dissociation of the axial histidine, with subsequent heme release and conformational changes. Although an SF experiment would rarely reach the minute timescale in practice, we used these artificially long experiments to be able to study the effects of UV–visible light illumination on the structural and spectroscopic properties of the enzyme. The onset of these effects is already in the millisecond timescale, which is relevant when using SF to study the mechanism of a heme enzyme. The UV–visible light-induced changes may obscure the observation of possible catalytic intermediates. The photosensitivity described here for *Ao*Cld may extend well to many other heme enzymes and, therefore, published pre-steady-state kinetic data should be re-evaluated and future kinetic studies using SF spectroscopy with white light and a photodiode detector should keep the photolabile nature of heme enzymes in mind.

## Availability of data

The datasets generated during and/or analysed during the current study are available from the corresponding author on reasonable request.

## Electronic supplementary material

Below is the link to the electronic supplementary material.

Supplementary file1 (PDF 577 kb)

## Abbreviations

*Ao*Cld: *Azospira oryzae* Chlorite dismutase
Cld: Chlorite dismutase
*C*Cld: *Cyanothece* Sp. PCC7425 chlorite dismutase
Cyt c: Cytochrome c
*Da*Cld: *Dechloromonas aromatica* Chlorite dismutase
EPR: Electron paramagnetic resonance
eq.: Equivalent
HRP: Horseradish peroxidase
IPTG: Isopropyl ß-d-1-thiogalactopyranoside
KPi: Potassium phosphate buffer
SEC: Size exclusion chromatography
SF: Stopped-flow
SVD: Singular value decomposition
