# Supplementary material for: Unexpected photosensitivity of the well-characterized heme enzyme chlorite dismutase
Source: J Biol Inorg Chem. 2020 Oct 28;25(8):1129–38. doi: 10.1007/s00775-020-01826-8 (PMC7665973; doi:10.1007/s00775-020-01826-8)
Supplement: Supplementary file 1 — Supplementary file1 (PDF 577 kb) [file 775_2020_1826_MOESM1_ESM.pdf]

Electronic supplementary information

Unexpected photosensitivity of the well-characterized heme enzyme chlorite dismutase

Durga Mahor[1], *Julia Püschmann*[1], *Diederik R. Adema*[1], *Marc J.F. Strampraad*[1], *Peter-Leon Hagedoorn*[1]*

[1]Department of Biotechnology, Delft University of Technology, Van der Maasweg 9, 2629HZ, Delft, The Netherlands

*Corresponding author
e-mail: p.l.hagedoorn@tudelft.nl
phone: +31-(0)15-2782334

ORCID P.L. Hagedoorn: 0000-0001-6342-2022

ORCID M.J.F. Strampraad: 0000-0003-1885-0604

Contents

1. Nucleotide sequence synthetic *Ao*Cld gene codon optimized for *E. coli* including N-terminal his-tag 2

2. Amino-acid sequence synthetic *Ao*Cld codon optimized for *E. coli* including N-terminal his-tag 2

3. Double exponential fit of the decay of the Soret peak at 402 nm of *Ao*Cld during the illumination experiment 2

4. Supplementary tables and figures 3

Fig. S1 UV-visible spectral changes of *Ao*Cld in a Cary60 UV-vis spectrophotometer. 3

Fig. S2 UV-visible spectral changes of *Ao*Cld with and without chlorite measured with stopped-flow (SF) spectroscopy 4

Fig. S3 The effect of catalytic turnovers on the photosensitivity of *Ao*Cld. 5

Table S1 Specific activity (U mg^{-1}) of illuminated and non-illuminated *Ao*Cld 6

Fig. S4 Size exclusion chromatography (SEC) calibration 6

Table S2 Molecular weight estimation of *Ao*Cld 6

1. Nucleotide sequence synthetic *Ao*Cld gene codon optimized for *E. coli* including N-terminal his-tag

5’ATGGGCAGCAGCcatcatcatcatcatcaCAGCAGCGGCCTGGTGCCGCGCGGCAGCCAT
ATGCAACCGATGCAGGCGATGAAAATTGAACGCGGAACAATACTGACGCAACCGGGTGTCTT
TGGCGTTTTTACAATGTTCAAATTACGCCCGGATTGGAACAAAGTGCCAGCAATGGAACGTA
AGGGTGCCGCTGAAGAAGTTAAGAAATTGATTGAGAAGCATAAGGATAACGTTCTGGTGGAC
CTCTACCTGACACGTGGCCTGGAAACCAATTCCGACTTTTTCTTCCGCATCAACGCGTACGA
CTTGGCAAAAGCCCAGACCTTTATGCGCGAATTTCGCTCGACTACTATCGGTAAAAATGCTG
ATGTGTTCGAGACCCTTGTCGGTGTCACAAAACCCTTGAATTACATCAGCAAAGACAAGTCT
CCTGGGCTGAATGCGGGACTTAGTTCAGCGACCTATAGTGGGCCGGCTCCACGGTATGTGAT
AGTAATTCCCGTCAAAAAGAACGCGGAATGGTGGAACATGTCGCCGGAAGAGCGTCTGAAAG
AGATGGAAGTTCATACGACACCTACCTTAGCTTATCTGGTGAATGTCAAACGCAAGTTGTAT
CACTCAACGGGCTTAGATGACACGGATTTCATTACCTATTTTGAAACTGATGATCTGACCGC
GTTTAACAACTTAATGCTGTCTCTGGCACAGGTTAAAGAGAATAAGTTTCACGTGCGTTGGG
GTAGCCCAACTACGCTCGGCACCATTCATTCCCCGGAAGATGTGATCAAAGCCCTTGCCGAT
TAA-3’

2. Amino-acid sequence synthetic *Ao*Cld codon optimized for *E. coli* including N-terminal his-tag

MGSSHHHHHHSSGLVPRGSHMQPMQAMKIERGTILTQPGVFGVFTMFKLRPDWNKVPAMERK
GAAEEVKKLIEKHKDNVLVDLYLTRGLETNSDFFFRINAYDLAKAQTFMREFRSTTIGKNAD
VFETLVGVTKPLNYISKDKSPGLNAGLSSATYSGPAPRYVIVIPVKKNAEWWNMSPEERLKE
MEVHTTPTLAYLVNVKRKLYHSTGLDDTDFITYFETDDLTAFNNLMLSLAQVKENKFHVRWG
SPTTLGTIHSPEDVIKALAD

3. Double exponential fit of the decay of the Soret peak at 402 nm of *Ao*Cld during the illumination experiment

$$A402 = A_1 e^{-k_{decay,1} \cdot t} + A_2 e^{-k_{decay,2} \cdot t} + A_0$$

A_0 = 0.091 ± 0.079
A_1 = 0.196 ± 0.089
$k_{decay,1}$ = 0.26 ± 0.21 min^{-1} = (0.43 ± 0.35)· 10^{-2} s^{-1}
A_2 = 0.578 ± 0.075
$k_{decay,2}$ = 0.014 ± 0.007 min^{-1} = (2.3 ± 1.2)· 10^{-4} s^{-1}

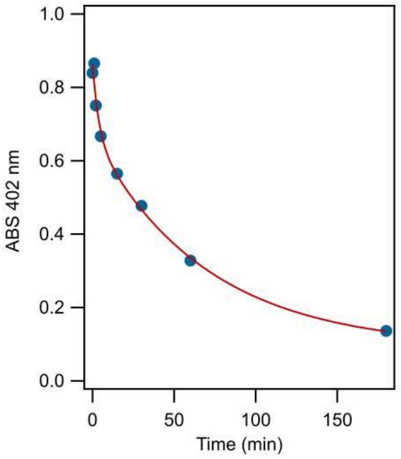

4. Supplementary tables and figures

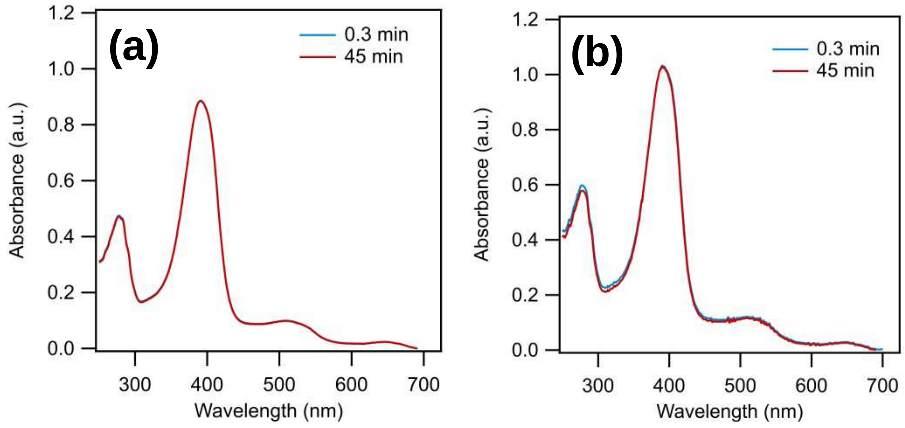

Fig. S1 UV-visible spectral changes of *Ao*Cld in a Cary60 UV-vis spectrophotometer.

The spectrophotometer contains a Xenon pulse lamp and a Czerny-Turner monochromator. Spectra were recorded every 0.3 min for 45 min at 21 °C while stirring. No significant change of the spectrum was observed for **a** 9.0 µM *Ao*Cld in 100 mM KPi pH 7.0 or **b** 11 µM *Ao*Cld in 50 mM KPi pH 7.0. Only the first and last spectrum are shown.

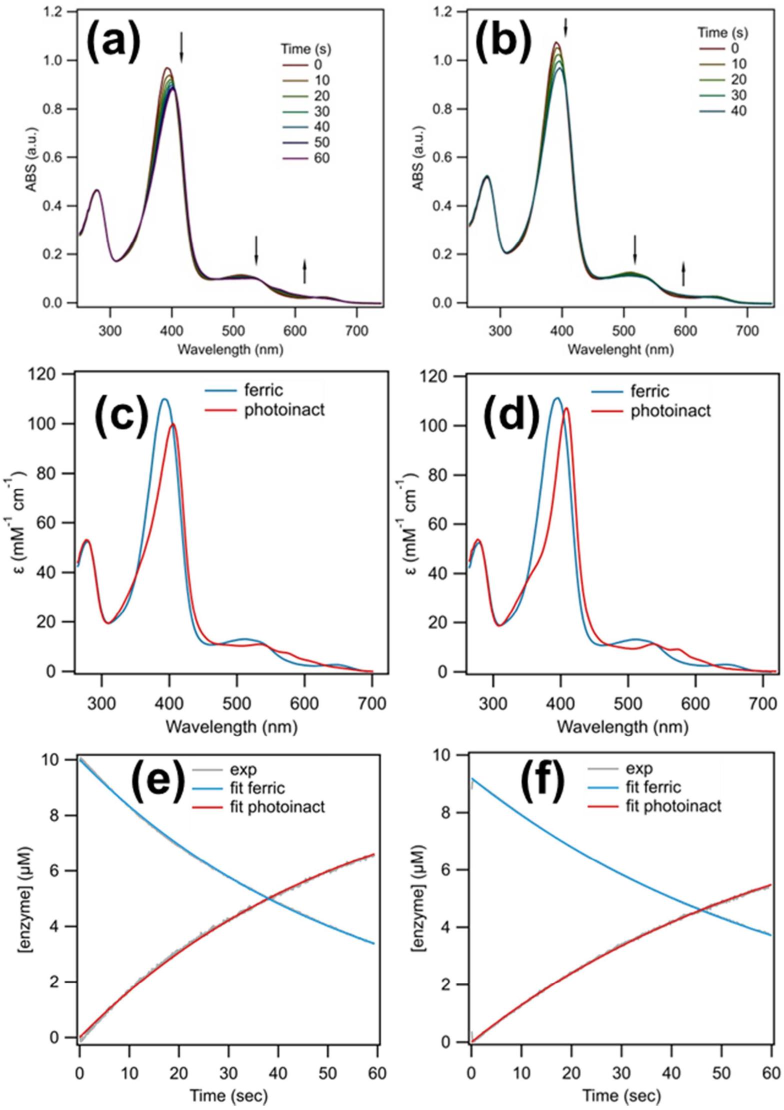

Fig. S2 UV-visible spectral changes of *Ao*Cld with and without chlorite measured with stopped-flow (SF) spectroscopy

a 20 µM *Ao*Cld in 100 mM KPi pH 7.0 was mixed (1:1) in the SF with water at 20 °C and spectra were recorded every 60 ms for 60 s. **b** 18 µM *Ao*Cld in 100 mM KPi pH 7.0 was mixed (1:1) in the SF with 20 µM chlorite in water at 20 °C and spectra were recorded every 60 ms for 60 s. **c** reconstructed spectra after SVD analysis of the SF data of *Ao*Cld versus water, in which ferric enzyme (blue) and photoinactivated species (red). **d** reconstructed spectra after SVD analysis of the SF data of *Ao*Cld versus 1 eq. chlorite, with ferric enzyme (blue) and photoinactivated species (red). **e** time traces of the two spectral species after SVD analysis (grey) of the *Ao*Cld versus water SF data and fits to first-order irreversible decay of ferric enzyme (blue) and increase of photoinactivated species (red) with k_1 = (1.82 ± 0.03) s^{-1}. **f** time traces of the two spectral species after SVD analysis (grey) of the *Ao*Cld versus 1 eq. chlorite SF data and fits to first-order irreversible decay of ferric enzyme (blue) and increase of photoinactivated species (red) with k_1 = (1.51 ± 0.05) s^{-1}.

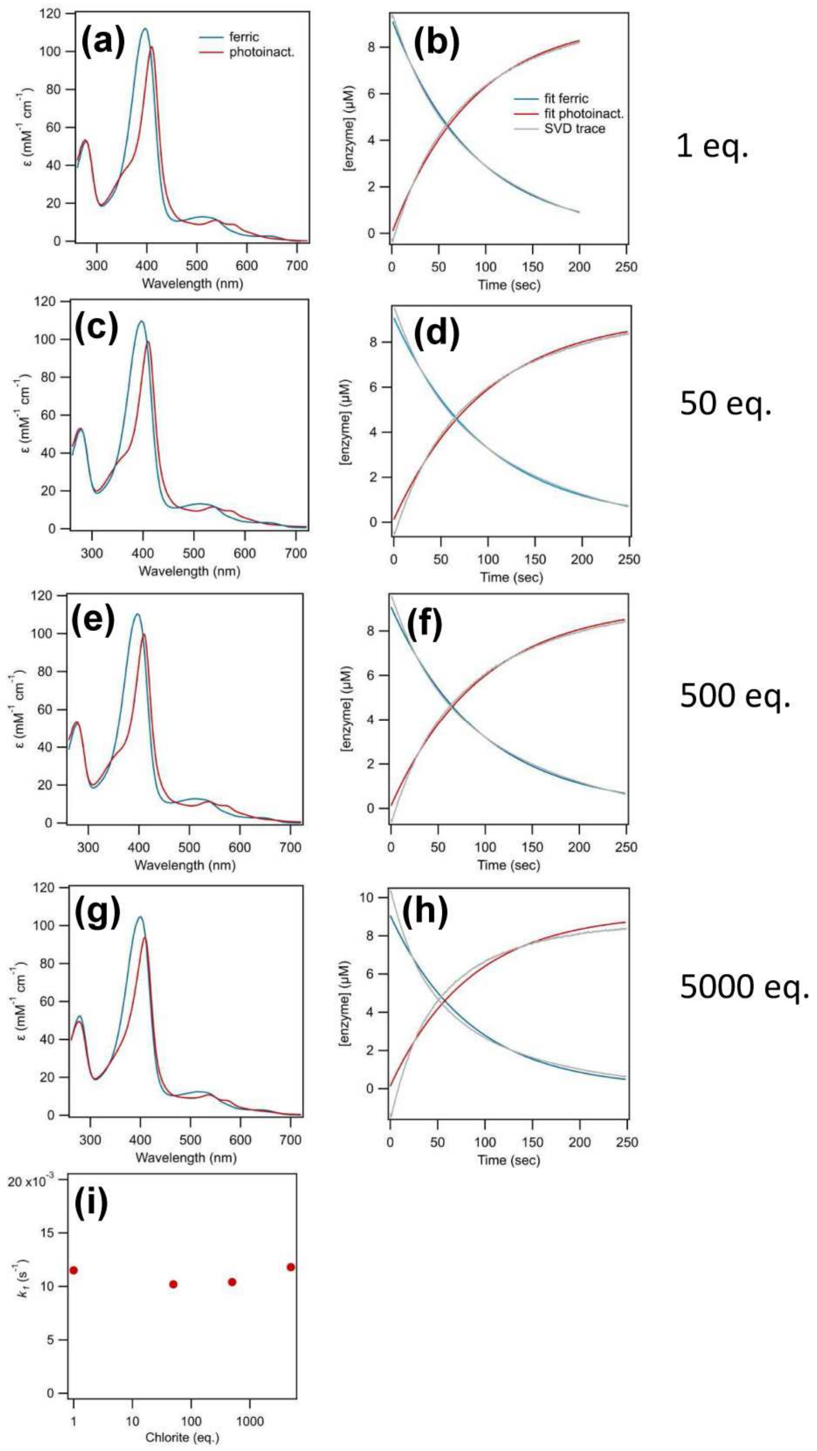

Fig. S3 The effect of catalytic turnovers on the photosensitivity of *Ao*Cld.

Kinetic analysis of SF spectroscopy experiments of 9 µM (final concentration) *Ao*Cld in 50 mM KPi pH 7.0 and different equivalents of chlorite at 20 °C and spectra were recorded every 0.2 s for 200 or 250 s. **a, c, e, g** reconstructed spectra after SVD analysis showing two species: ferric enzyme (blue) and photoinactivated species (red). **b, d, f, h** time traces of the two spectral species after SVD analysis (grey) and fits to first-order irreversible decay of ferric enzyme (blue) and increase of photoinactivated species (red). **i** first-order rate constant k_1 at different initial chlorite equivalents. In all cases the chlorite was completely consumed within less than 1 sec.

Table S1 Specific activity (U mg^{-1}) of illuminated and non-illuminated *Ao*Cld

Illumination time (min)	Specific activity (10^3 U mg^{-1})
Non-illuminated	8.53 ± 0.16
30 min	2.32 ± 0.05
60 min	1.99 ± 0.11
180 min	0.126 ± 0.006

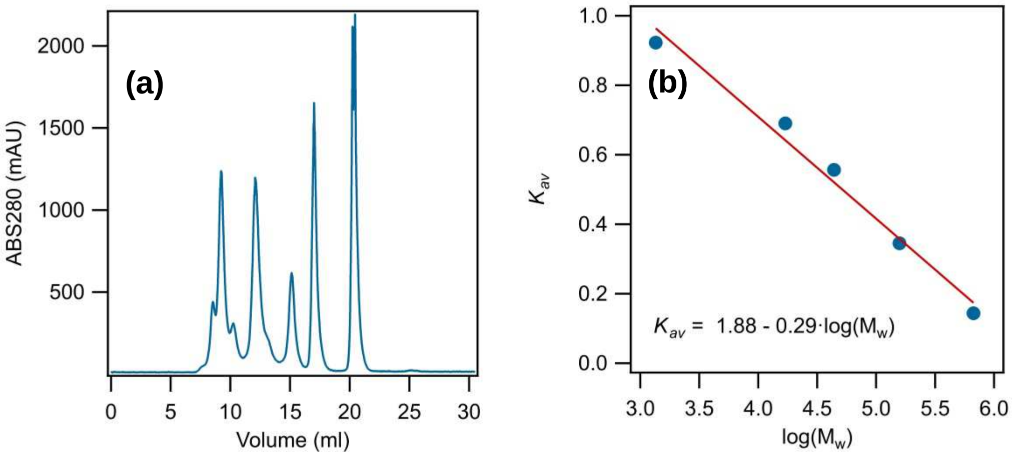

Fig. S4 Size exclusion chromatography (SEC) calibration

a SEC chromatogram of the protein standards. **b** SEC calibration curve. Protein standards SEC with Thyroglobulin (670 kDa), γ-globulin (158 kDa), Ovalbumin (44 kDa), Myoglobin (17 kDa) and Vitamin B_{12} (1.35 kDa). SEC conditions: column Superdex S200 10/300 GL, flow rate 0.5 ml/min, and running buffer 100 mM KPi pH 7.0.

Table S2 Molecular weight estimation of *Ao*Cld

Peak	V_e (ml)	K_{av}	Mw calculated (kDa)	Apparent oligomeric state
*Ao*Cld native, major peak	12.5	0.373	140	pentamer
*Ao*Cld illuminated, major peak	15.9	0.613	21.3	monomer
*Ao*Cld illuminated, minor peak	14.3	0.500	51.7	dimer

V_e is the elution volume. Void volume V_o = 7.2 ml, total column volume V_t = 21.4 ml. Gel-phase distribution coefficient $K_{av} = \frac{V_e - V_o}{V_t - V_o}$. The Mw of *Ao*Cld 30.3 kDa per subunit.
